# Supplementary material for: Highly structured, partner-sex- and subject-sex-dependent cortical responses during social facial touch
Source: Nat Commun. 2019 Oct 11;10:4634. doi: 10.1038/s41467-019-12511-z (PMC6789031; doi:10.1038/s41467-019-12511-z)
Supplement: Supplementary file 1 — Supplementary Information [file 41467_2019_12511_MOESM1_ESM.pdf]

Supplementary Information

Highly structured, partner-sex– and subject-sex-dependent cortical responses during social facial touch
Ebbesen et al., 2019

This PDF file includes:
Supplementary Figures 1-7
Supplementary Notes 1-2

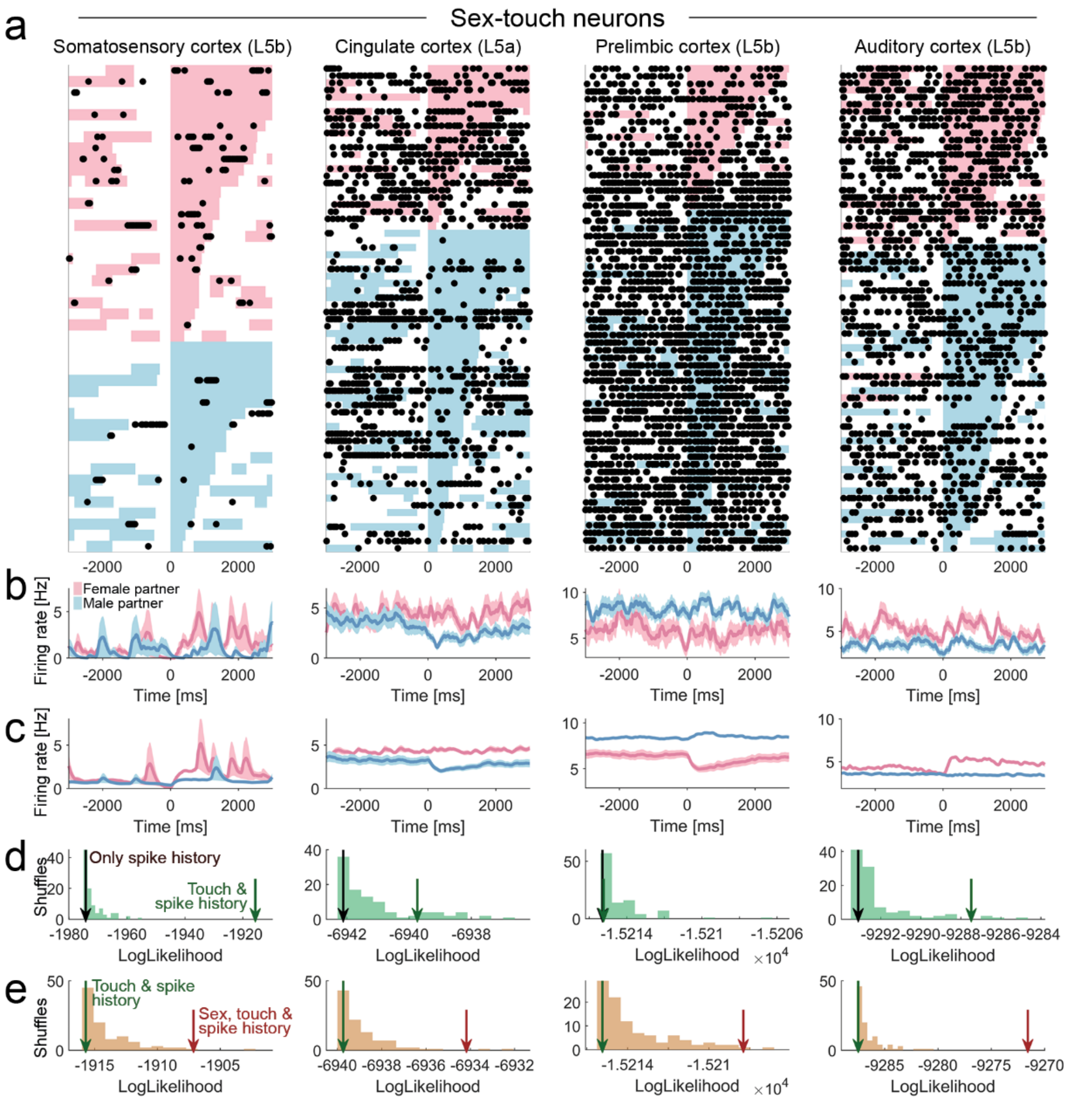

Supplementary Figure 1 I More example sex-touch neurons from the other brain areas. (a) Raster plot of example sex-touch neurons for the brain areas not shown in Figure 2 (an S1 L5b neuron, an ACC L5a neuron, a PrL L5b neurons and an A1 L5b neuron). Raster plots show spike times (black dots) aligned to the first whisker-to-whisker touch in each social touch episode. Social touch episodes are sorted by partner sex (female: pink, male: blue) and by duration (indicated by length of colored bar). (b) Peri-stimulus time histograms of the example neurons shown in (a), separated by partner sex. Black line indicates mean firing rate, smoothed with a Gaussian kernel (σ = 100 ms), shaded area indicates s.e.m, pink/blue color indicates female/male partner animals. (c) Peri-stimulus time histograms of the example neurons shown in (a), calculated from the fitted regression model (plot conventions as in (b)). (d) Estimating touch-modulation: Log-likelihood values of models fitted to the neurons in (a). The log-likelihood of models depending on touch is indicated by a green arrow, the log-likelihood of models without touch is indicated by a grey arrow and the log-likelihood distribution of shuffled touch-models is indicated by green bars. Some sex-touch neurons would not be significant, if the partner sex was not considered in the model. For example, the ACC L5a neuron is suppressed by males, but shows (almost) no response with females, so the green arrow is not in the 0.05 fraction of the shuffled distribution if all touches are pooled, without parsing out the partner sex. (e) Estimating sex-touch-modulation: Log-likelihood values of models fitted to the neurons in (a). The log-likelihood of models depending on partner sex and touch is indicated by a brown arrow, the log-likelihood of model without sex is indicated by a green arrow and the log-likelihood distribution of shuffled sex-touch-models is indicated by brown bars. All neurons are significant at $p < 0.05$ (brown arrow outside the shuffled distribution).

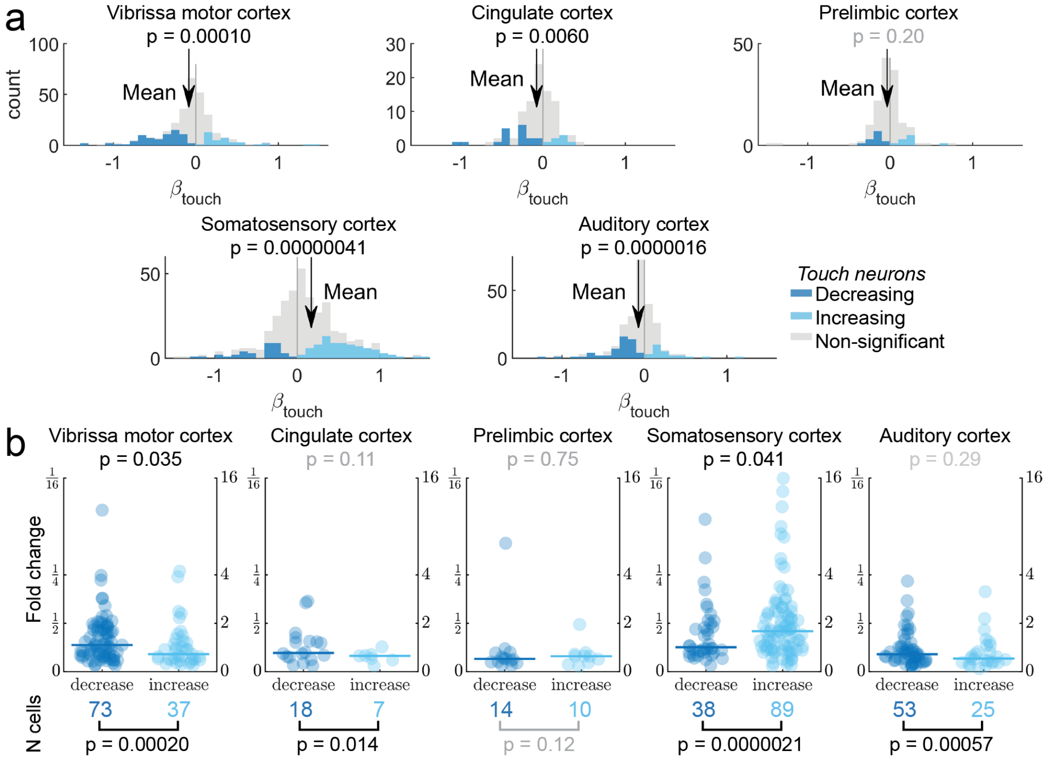

Supplementary Figure 2 | Prototypical responses during social touch vary by cortical area. (a) Distribution of fitted β_{touch} across cortical areas (axis clipped at +/–1.6, all data used for calculations, all data plotted in S2A-B). Colored bars indicate significantly increased neurons (light blue), significantly decreased neurons (dark blue) and non-significant neurons (grey). Black arrow indicates mean β_{touch}, p-value indicates Wilcoxon signed rank test. As a population, S1 neurons increased in firing rate during social touch (mean β_{touch} = 0.17, p = 0.00000041, N = 384, Wilcoxon signed-rank test). VMC, ACC and A1 neurons decreased (VMC/ACC/AC: mean β_{touch} = –0.084/–0.077/–0.073, p = 0.00010/0.0060/0.0000016, N = 296/95/239, Wilcoxon signed-rank test) and PrL neurons did not show any modulation at the population level (mean β_{touch} = –0.036, p = 0.20, N = 142, Wilcoxon signed-rank test). (b) *Top:* Fold change in firing rate by social touch for significantly increased (light blue) and decreased neurons (dark blue). Horizontal lines indicate medians, p-values indicate Mann-Whitney U-test. *Below:* Number of neurons, which are significantly increased (light blue) and decreased (dark blue) by social touch. P-values indicate binomial test. In S1, more neurons were significantly increased by touch (decreasing v. increasing neurons, 38 v. 89, p = 0.0000021, binomial test) and the significantly increasing neurons were the most strongly modulated neurons (decreasing v. increasing neurons, median |log2(ratio)| = 0.51 v. 0.84, p = 0.041, Mann-Whitney U test). In VMC, more neurons were decreasing (73 v. 37 neurons, p = 0.00020, binomial test) and the decreasing neurons were most strongly modulated In VMC (median |log2(ratio)| = 0.55 v. 0.36, p = 0.035, Mann-Whitney U test). ACC and A1 both had more significantly decreasing neurons (ACC: 18 v. 7 neurons, p = 0.014, AC: 53 v. 25, p = 0.00057, binomial test), but no significant difference in the modulation strength. (ACC: median |log2(ratio)| = 0.39 v. 0.33, p = 0.11, A1: median |log2(ratio)| = 0.36 v. 0.27, p = 0.29, Mann-Whitney U-test). PrL had a similar number of increasing and decreasing neurons and no differences in modulation strength (14 v. 10 neurons, p = 0.12, binomial test, median |log2(ratio)| = 0.26 v. 0.32, p = 0.75, Mann-Whitney U test).

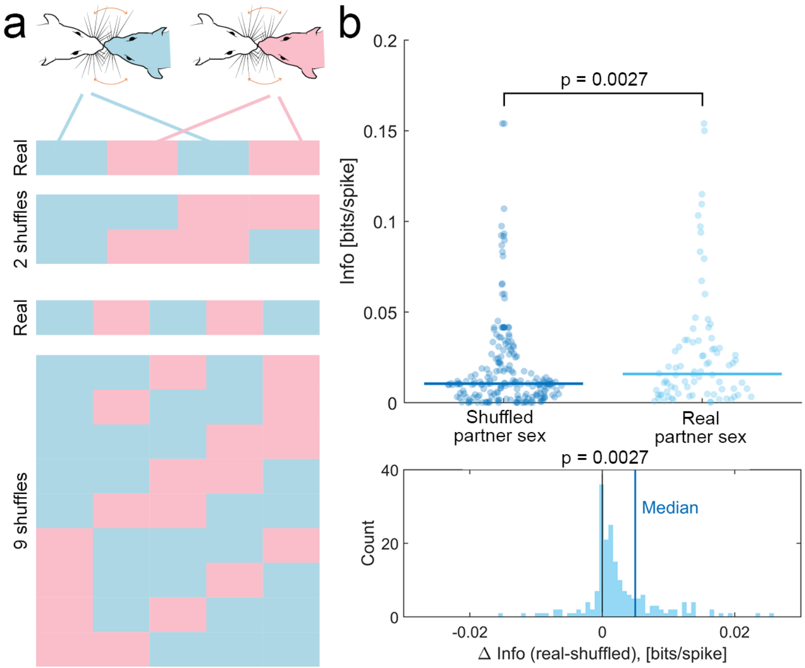

Supplementary Figure 3 I Partner sex patterns responses more than individual identity. If neurons did not encode the sex of the stimulus animals at all, but had individual-specific responses, we would expect to sometimes identify artefactual 'sex-touch' neurons, simply because we are comparing two groups of animals with individual responses. Since we only presented few partner animals, it is difficult to ask (at the single-cell level) if responses of these neurons are individual-specific or sex-specific. One way to ask if ostensibly partner-sex-dependent response differences are indeed driven by the sex of the partner animal is to ask if grouping the responses to partner animals by their real sex is more informative about spike rates, than grouping the responses to partner animals by a shuffled sex. We frame the analysis in terms of mutual information between spike rates, because this allows us to be agnostic about the direction of modulation (increases/decreases). First, we used a shuffling procedure to identify neurons, most likely to carry individual-specific information (see Methods). The number of possible shuffles depends on the number of partner animals in the particular recording session (typically 2 males and 2 females, panel a). To overcome this imbalance in the data, we used a mixed-effects modeling approach[9] (panel b). We found that putatively individual-specific neurons were significantly more informative about sex than all other possible partitions of the data (mean ΔInfo = 0.007 bits/spike, $p = 0.0027$, mixed-effects model, panel b). This analysis does not exclude the possibility that some neurons with individual-specific responses might still be present in these brain areas. However, it shows that the sex of the partner animal is indeed a major determinant of the firing patterns, and that partner-sex-specific modulation is not an artifact better explained by individual-specific effects.
(a) Number of possible shuffles grows with number of interaction partners. If an animal interacted with two male and two female conspecifics, we can only generate two possible shuffled assignments of the partner sex (top). If the animal interacted with three males and two females, we can generate nine possible shuffled assignments of the partner sex (bottom). (b) Top: Distribution of information per spike calculated using real (light blue) and shuffled (dark blue) assignments of partner sex. Below: Distribution of difference between real and shuffled information per spike (p-value indicates mixed-effects model, see Methods).

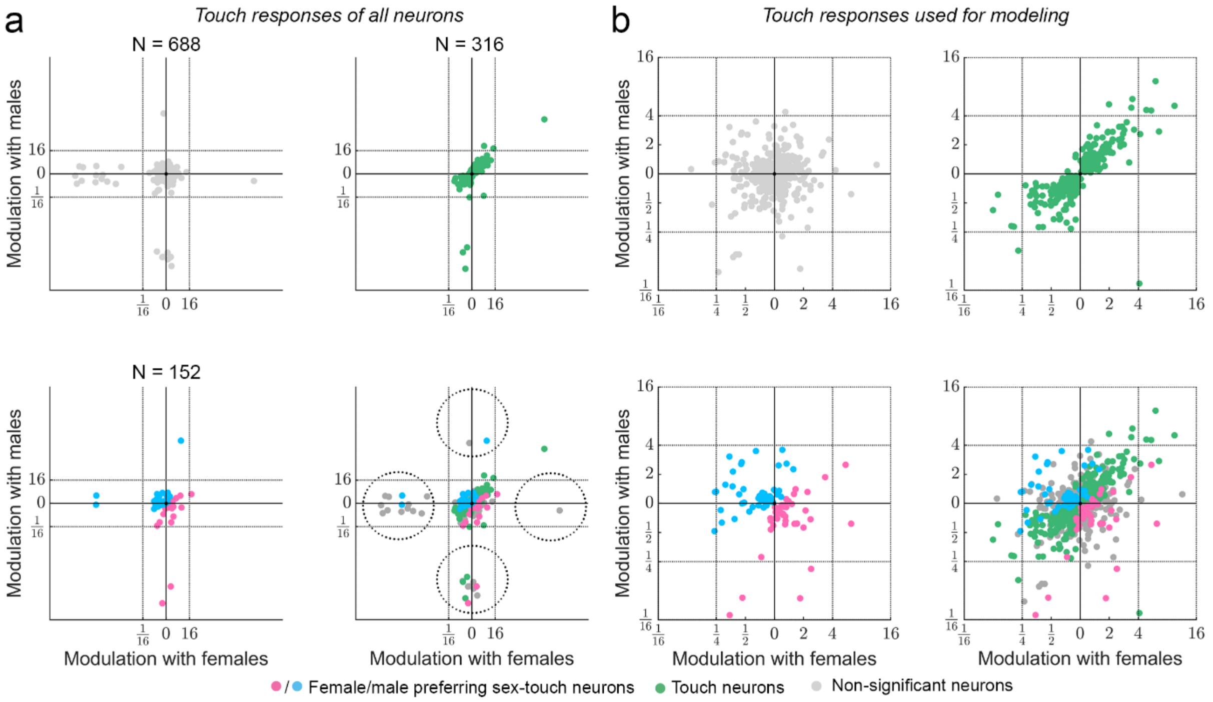

Supplementary Figure S4 I Population response patterns across all areas. (a) Modulation of activity (in fold change) of all neurons during social touch with male and female conspecifics. Touch neurons are indicated by green dots, female/male preferring sex-touch neurons are indicated by pink/blue dots and non-significant neurons are indicated by grey dots. Since the statistical modeling of the spike train models all modulation as ratios, a few neurons with either very low baseline firing rates, or which were essentially silenced during touch will be fitted to very high/low values of modulation (indicated by dotted circles). (b) Same plots as (a), but zoomed in to only show neurons between 16-fold increase and 16-fold decreases in firing rate (the vast majority of neurons). In order not to skew the GLM models by the extreme outliers, we only used neurons with less than 32-fold modulation in the GLM models. Touch neurons are indicated by green dots, female/male preferring sex-touch neurons are indicated by pink/blue dots and non-significant neurons are indicated by grey dots.

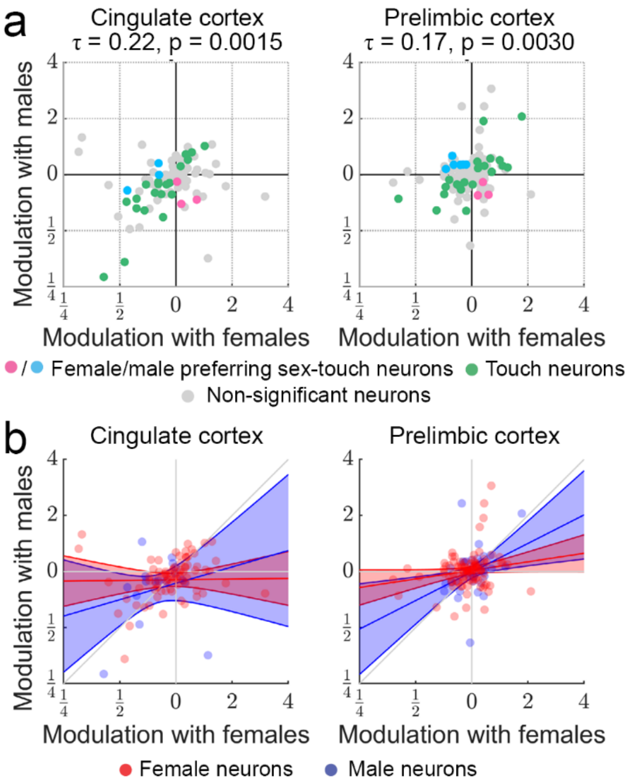

Supplementary Figure 5 l Population response patters during social facial touch for cingulate and prelimbic cortex. (a) Same plot as Figure 4b, but showing data from ACC and PrL. Modulation of activity (in fold change) during social touch with male and female conspecifics is highly correlated. Touch neurons are indicated by green dots, female/male preferring sex-touch neurons are indicated by pink/blue dots and non-significant neurons are indicated by grey dots, Kendall's τ and p-value above. (b) Same plot as Figure 5b, but showing data from ACC and PrL. Although not significant, the maximal-likelihood fit also estimated $\beta_{subject\_sex}$ to be less than unity for both ACC and PrL. This pattern is in line with the pattern in S1, VMC and AC (Figure 4d). Red/blue dots indicate neurons recorded in female/male subject animals, red/blue lines indicate maximum-likelihood fit of regressing modulation with males as a function of modulation with females, for female/male subjects (see Methods and Supplementary Note 2 for model specification), shaded area indicates 95% C.I.

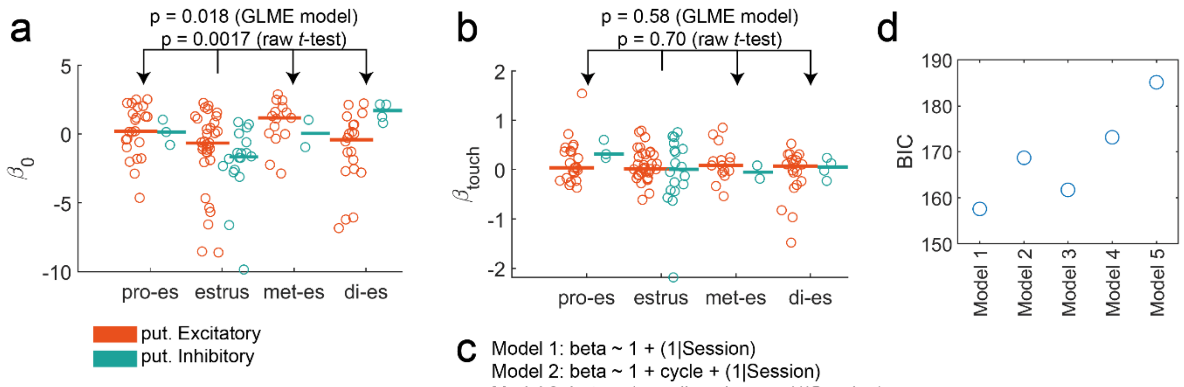

c Model 1: beta ~ 1 + (1|Session)
Model 2: beta ~ 1 + cycle + (1|Session)
Model 3: beta ~ 1 + spike_shape + (1|Session)
Model 4: beta ~ 1 + cycle + spike_shape + (1|Session)
Model 5: beta ~ 1 + cycle + spike_shape + cycle*spike_shape + (1|Session)

Supplementary Figure 6 l Responses to social facial touch in somatosensory cortex do not depend on the estrus state. (a) In a subset of the S1 data from female subject animals, we had access to the estrus state at the day of the experiment. In agreement with ref. [4] (same data) and ref. [10], we found that firing rates (quantified here as β_0, so baseline rate $r_{base} = \exp(\beta_0)$) were significantly lower during estrus than non-estrus. We did two tests: a "raw" t-test where we pooled all non-estrus states (like in ref. [4]) and tested against the estrus state cells (p = 0.0017), and a more sophisticated model, where we treat all non-estrus days (pro-estrus, met-estrus and di-estrus) as independent categorical variables, control for unequal number of neurons recorded on the same experimental session, fit a GLME regression and do the full ANOVA (p = 0.018). Both were significant. For plotting, we have split putative excitatory (orange) and inhibitory (teal) neurons, vertical lines indicate medians. (b) Same analysis as in (a), but here we analyze the responses to social touch (quantified as β_{touch}). We did not find any differences in response magnitude across the estrus cycle (neither using a pooled *t*-test or the GLME model: p >> 0.05, same finding as ref. [10]). (c) We tried a variety of GLME models to see if responses depended on estrus state ('cycle', 4 levels) or putative inh./ex. neuron type ('spike_shape', 2 levels, shown in Supplementary Figure 7), or both (interactions), but none of these models had significant effects or did better than a constant model (models compare by Bayesian information criterion, model specification in Wilkinson notation). (d) Bayesian information criteria (BIC) for the models shown in (c). The constant model ('Model 1') has the lowest BIC.

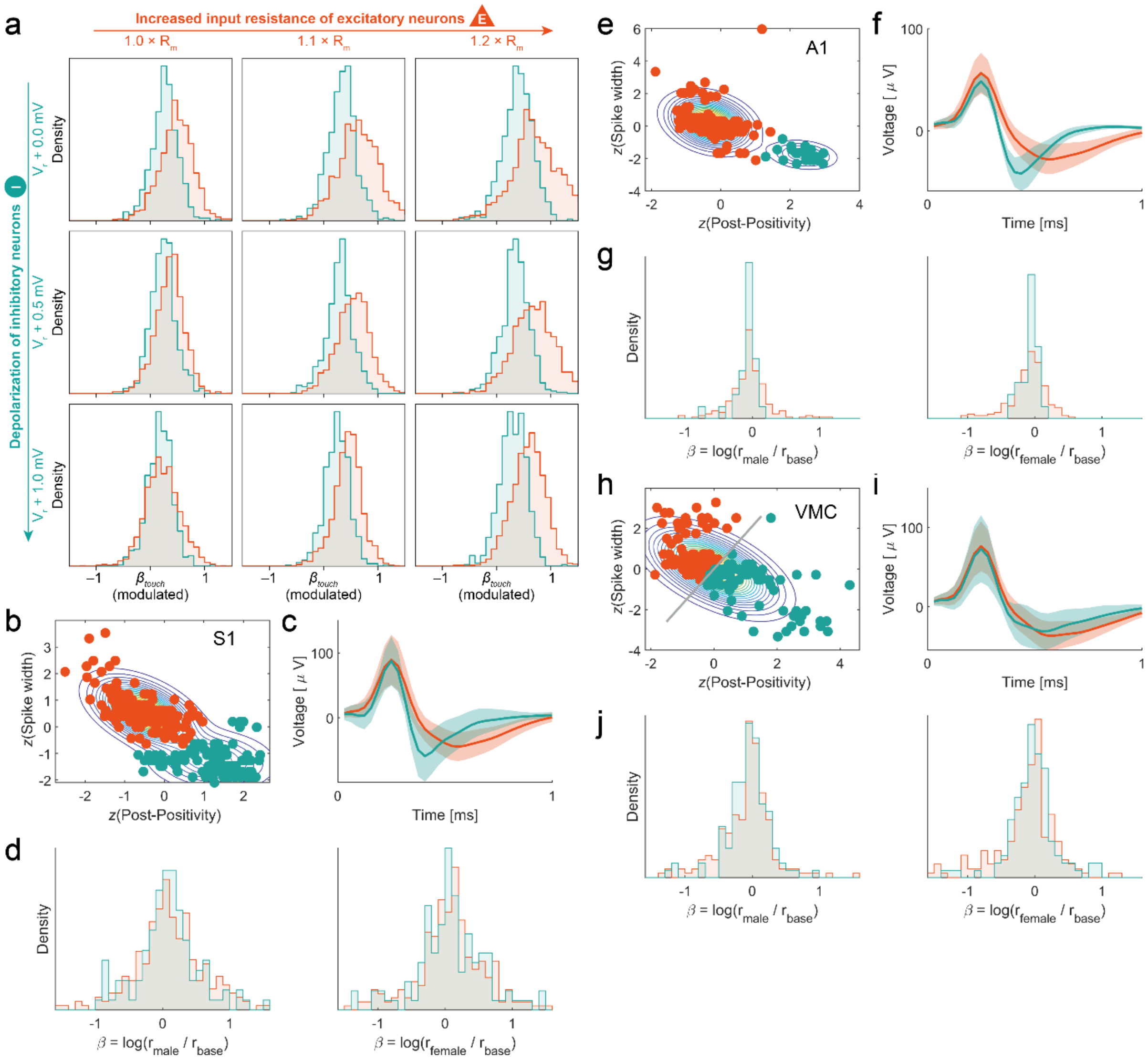

Supplementary Figure 7 | Putatively inhibitory and excitatory neurons respond in the same direction and overlap, consistent with simulated responses to 'touch' (thalamic input). (a) Density of responses during touch of simulated excitatory (orange) and inhibitory (teal) neurons are in the same direction an overlap (more and more strongly, going down along first column, same plotting conventions as Figure 5c). (b) We separated putatively excitatory (orange) and inhibitory (teal) neurons recorded in somatosensory cortex by two features: the spike width (at half maximum) and the 'post-positivity' (the integral of the spike waveform between 0.375 ms and 0.75 ms after the spike peak, normalized by peak voltage). We assigned the neurons two clutsers by z-scoring the features, fitting a two-component Gaussian distribution and assigning the neurons by the component yielding the highest posterior probability (see Methods). (c) Mean spike shape of putatively excitatory (orange) and inhibitory (teal) neurons recorded in somatosensory cortex (shaded area indicates standard deviation).(d) Density of responses during social touch to male and female conspecifics of putatively excitatory (orange) and inhibitory (teal) neurons recorded in somatosensory cortex. (e-g) Same as (b-d), but for neurons recorded in auditory cortex. (h-j) Same as (b-d), but for neurons recorded in vibrissa motor cortex. Note in panel h, the spike widths and post-positivity did not form a bimodal distribution, so we just fitted a single multivariate Gaussian and 'cut' that gaussian in two along the shortest axis (grey line, please see Methods).

Supplementary Note 1: *A brief introduction to β-coefficients as a metric of firing rate changes*

In this study, we want to investigate how social touch and social context impact the firing patterns of cortical networks. Thus, we need a metric which allows us to quantify changes in firing rate of both single neurons and populations of neurons. In this short note, we explain why using regression coefficients ("β-coefficients") as metric is a natural choice. We also compare the β-coefficients to other commonly used metrics of firing rate change.

Not all metrics are suitable for quantifying patterns in population activity
Let us first consider a 'classic' peri-stimulus time histogram (PSTH). For a cartoon neuron, a PSTH aligned to the beginning of social touch might look like this:

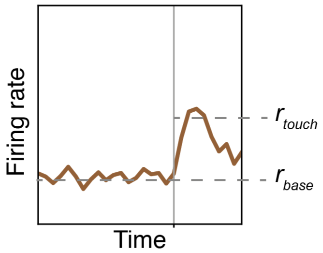

Which metric should we use to quantify how much the firing rate changes during touch? One possibility would be to estimate a baseline firing rate (r_{base}) and a firing rate during touch (r_{touch}) and simply measure the change in firing rates: $\Delta r = r_{touch} - r_{base}$. This metric does not take the baseline firing rate into account, and it is thus difficult to compare across neurons. For example, for these two cartoon neurons, Δr is the same, but it seems right to say that the neuron on the left is more "strongly modulated" than the neuron on the right:

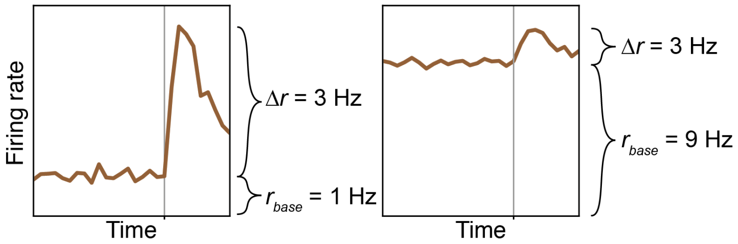

The z-score is another commonly used metric of firing rate changes. In a PSTH-based analysis, the z-score measures how much the firing rate during touch increases above the baseline firing rate, measured in multiples of the standard deviation of the baseline firing rate:

$$z = \frac{r_{touch} - r_{base}}{\sigma(r_{base})}.$$

Like Δr, the z-score does not normalize by the baseline firing rate and it is thus also difficult to compare z-scores across neurons. Moreover, since z-scores are normalized by the standard deviation of the baseline firing rate, z-scores of a PSTH depends on the number of trials. For example, even though these two cartoon neurons respond equally to touch, the left neuron will have a small z-score (there are few trials, so the PSTH is noisy and has a high standard deviation), whereas the right neuron will have a large z-score (there are many trials, so the baseline firing rate is very smooth and flat):

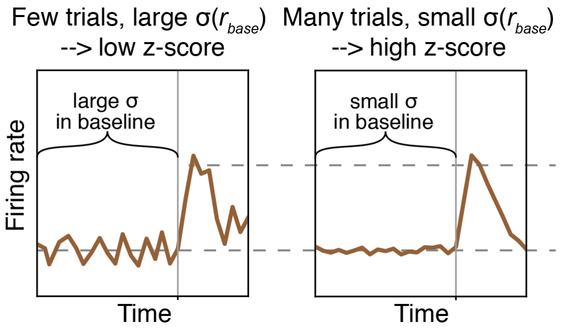

We need a metric which takes baseline firing rates into account

Clearly, both Δr and z-scores are not good metrics if we want quantify the modulation of a whole population of neurons. We need a metric that normalizes the change in firing rate by the baseline firing rate. Let us consider three options:

1. One option is to quantify the modulation a neuron using the metric $k = \frac{\Delta r}{r_{base}}$. The interpretation of this metric is simply the magnitude of change measured in units of the baseline rate. Firing rates cannot be negative ($r \leq 0Hz$), so this metric takes values in the interval $[-1, \infty[$. For neurons with decreasing firing rates, k will be between –1 and 0, whereas for neurons with increasing firing rates, k can be arbitrarily large.

2. A second option would be to quantify modulation using the metric $a = \frac{r_{touch}}{r_{base}}$. The interpretation of this metric is as the ratio of firing rate during touch and baseline. This metric takes values in the interval $[0, \infty[$. For neurons with decreasing firing rates, a will be between 0 and 1, whereas for neurons with increasing firing rates, a can be arbitrarily large.

3. A third option is to wrap the ratio of firing rates inside some function. Any function could be used in our metric, as long as that function is injective (every $f(a)$ is unique for all possible values of a). For example, we can choose the logarithmic function, and define our metric as $\beta_{touch} = log(a) = log(\frac{r_{touch}}{r_{base}})$. Because we used the logarithm, the interpretation of the metric β_{touch} is straightforward: β_{touch} is a 'fold change'. This metric takes values in the interval $]-\infty, \infty[$[1]. In contrast to k and a (where increases in firing rates lead to arbitrarily large values of the metric, but decreases in firing rates were assigned values between –1 and 0, or 0 and 1), β_{touch} is symmetric around 0. Doubling the firing rate during touch ($a = 2$) and halving the firing rate during touch ($a =$½) yield β_{touch} of equal magnitude, because both are a "two-fold" change. Since we used the natural logarithm, $\beta_{touch} = \pm 1$ corresponds to an e-fold increase and an e-fold decrease, respectively:

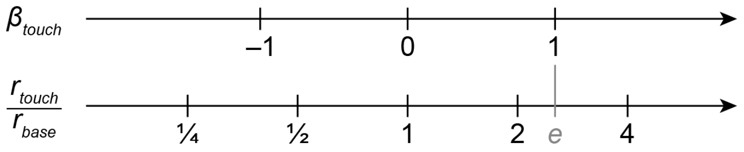

[1] Strictly speaking, if either the baseline or the touch rate is 0 Hz, log(a) is not defined, but tending to positive and negative infinity, respectively. This matches our intuition since, for example, total cessation of spiking is an "infinitely strong" decrease.

What are the reasons to prefer one metric over the other?

We have introduced three metrics, which quantify how the firing rates of single neurons change during episodes of social touch:

"Delta over baseline" "Ratio" "Fold increase"

$$\frac{\Delta r}{r_{base}} \qquad \frac{r_{touch}}{r_{base}} = \frac{\Delta r}{r_{base}} + 1 \qquad \beta_{touch} = \log\left(\frac{r_{touch}}{r_{base}}\right)$$

These metrics all measure the same thing and one can be calculated from the other, so what is the reason to prefer one over the others? The reason is the shape of their distribution across the network. In this study, we want to quantify patterns in the activity in large populations of neurons, and ask how these patterns depend on sex of the partner animal and sex of the subject animal. Such questions require analysis of the variance of our metric. Generalized linear mixed-effect modeling (GLME models) is a powerful statistical method that allows us to perform such analysis of variance (ANOVA), but such regression methods are only valid if we can model the error distributions. Specifically, if the errors follow a Gaussian distribution, or at least an approximately Gaussian distribution, we can use GLME modeling to analyze how touch responses depend on partner sex and subject sex, while simultaneously controlling for the fact that we have an unequal number of neurons from the experimental subject animals[9].

So which metric is a natural choice for cortical firing rates? That is an empirical question. A first clue is provided by the fact that baseline firing rates across neural populations are usually log-normally distributed[13]. This is also the case in our dataset. The distribution of firing rates in our data is heavily skewed towards low firing rates with a heavy tail of few, high-firing neurons. If we plot the distributions of firing rates on a logarithmic axis, the distribution is approximately Gaussian (best Gaussian fit is plotted on top):

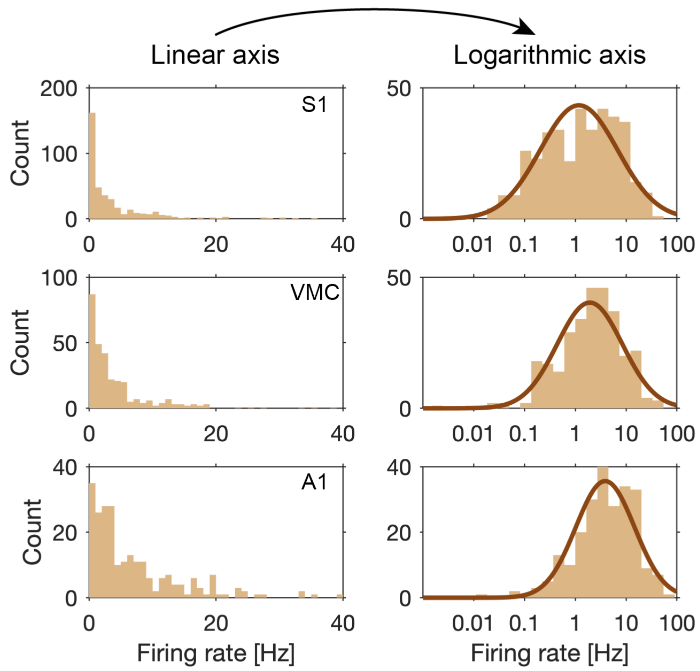

Since firing rates are log-normally distributed, it seems likely that changes in firing rates also follow a log-normal distribution. However, neurons are non-linear units of computation that utilize a wealth of nonlinear synaptic, somatic and dendritic mechanisms to transform synaptic input into output firing[14], so weather changes follow a log-normal distribution is an empirical question which we should check. As expected, we find that also *changes* in firing rates during touch are log-normally distributed (see also Fig. S3a). If we plot our three metrics for changes in firing rate during social touch in somatosensory cortex, for example, we can

see that the distributions of $\frac{\Delta r}{r_{touch}}$ and $\frac{r_{touch}}{r_{base}}$ are skewed and not bell-shaped (they have a high skewness and a Gaussian distribution is a poor fit with a high negative log-likelihood). In contrast, the distribution of β_{touch} is approximately normal (the distribution has a low skewness and Gaussian distribution is a good fit, with a much lower negative log-likelihood):

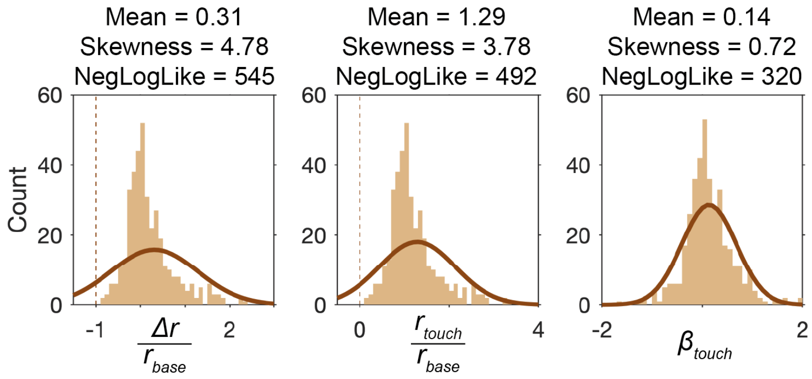

Ok, so both firing rates and *changes* in firing rates are approximately log-normally distributed. This is nice, since if we talk about an average β_{touch}, for example, we are talking about a meaningful quantity, the mean of a bell-shaped distribution. The observant reader will note, however, that for generalized linear modeling, it's not actually the variables, but rather the residuals of the variables that have to be normally distributed. So how do the residuals look? In our modeling, we want to model how the change in firing rates when touching female partners and touching male partners differs. If we plot both $\frac{\Delta r}{r_{touch}}$, $\frac{r_{touch}}{r_{base}}$ and β_{touch} for male and female partners (as in Fig 4. c-d) and fit a linear regression (top row below), they all suggest the same direction of the effect. However, when we inspect the residuals, we find that the regression residuals for $\frac{\Delta r}{r_{touch}}$ and $\frac{r_{touch}}{r_{base}}$ are quite skewed (Skewness > 1, meaning that assumptions of the the statistical model are violated), whereas the regression residuals for β_{beta} are approximately normal (meaning that the statistical model is valid).

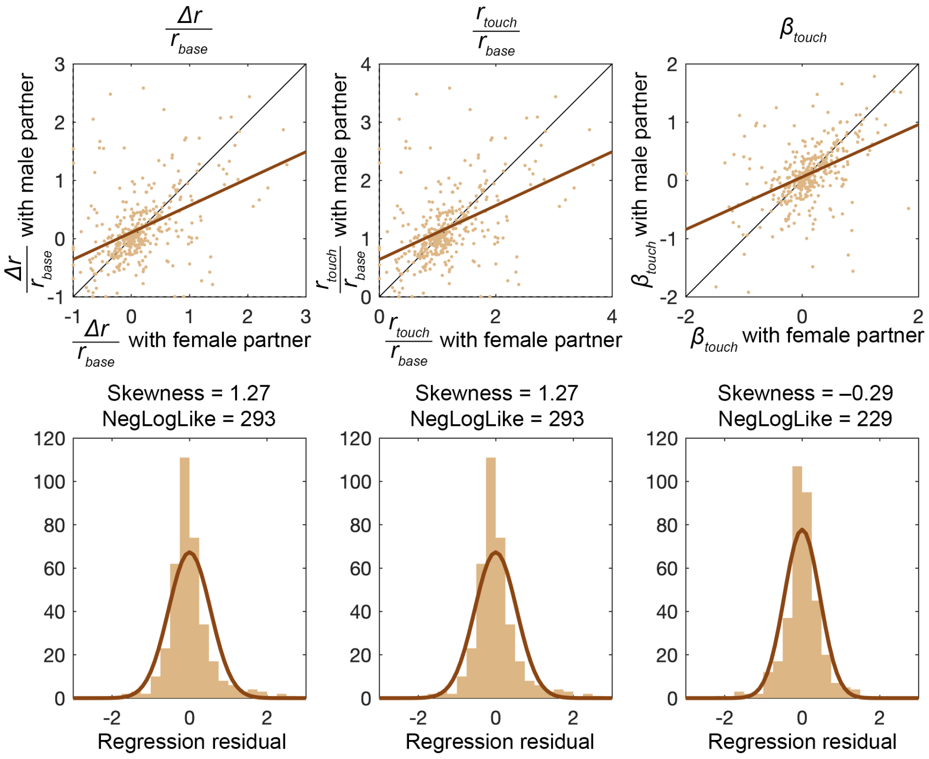

In summary, since both firing rates and changes in firing rates are log-normally distributed across cortical populations, β_{touch} is a natural metric for the network (the distribution is normal, so the mean and standard deviation is meaningful). Moreover, since the residuals of β_{touch} are approximately normal, statistical models relying on normally distributed errors are valid and we can use statistical modeling to quantify how the network firing patterns change with both social touch and social context.

What is the connection to the regression model?
In our spike train regression, we model the firing rate of single neurons as a depending on spike history, recording session (to allow for baseline drift), touch and partner sex (see Methods). We assume that these effects are independent of each other, so we express the influence of the covariates on the instantaneous firing rate, r, by expressing the firing rate as a product of functions of the covariates[5]:

$$r = constant \cdot f(spike\ history) \cdot f(recording\ session) \cdot f(touch) \cdot f(sex)$$

We are free to choose any functions f, as long as $f \geq 0$ (firing rates cannot be negative) and f is injective (every $f(x)$ is unique). The exponential function is a natural and convenient choice ($e^x \geq 0$ for all x and e^x is injective). Moreover, exponential functions are convenient for computational reasons, since to calculate products of exponential functions, we just have to sum their exponents. In our model, touch and sex are binary variables, which we code as 0 and 1. In order to scale the relative impact of the various covariates, we need scaling factors, which we can pack inside the exponential function and call "β". For example, if we disregard spike history, recording session and sex for now, we could write:

$$r = constant \cdot f(touch) = exp(\beta_0) \cdot exp(\beta_{touch} \cdot touch)$$

Touch is coded as a binary variable (0 or 1) so after we have used likelihood maximization to estimate the β-coefficients, we can calculate the baseline firing rate and the firing during touch as:

$$r_{touch} = exp(\beta_0) \cdot exp(\beta_{touch} \cdot 1) = exp(\beta_0 + \beta_{touch})$$
$$r_{base} = exp(\beta_0) \cdot exp(\beta_{touch} \cdot 0) = exp(\beta_0)$$

From this formulation, we can see that β_{touch} is the same as we introduced above:

$$\frac{r_{touch}}{r_{base}} = \frac{exp(\beta_0 + \beta_{touch})}{exp(\beta_0)} = exp(\beta_{touch})$$
$$\Updownarrow$$
$$\beta_{touch} = log\left(\frac{r_{touch}}{r_{base}}\right)$$

Calculating responses with male and female interaction partners
In our modeling, we code periods of social touch as 0 and 1. If we do not model the effect of sex, then we can calculate the maximum likelihood estimate of the baseline and touch as shown above. In the models where we also fit the effect of sex, we code the data with an indicator variable such that $sex = 1$ corresponds to touching as male and $sex = 0$ corresponds to touching a female. In that way, we can fit a model where touch episodes with male and female conspecifics lead to different firing rate changes. If we again disregard spike history effects and baseline drift between recordings, we can calculate the firing rate when touching male and female conspecifics like this:

$$r_{touching\ a\ male} = exp(\beta_0) \cdot exp(\beta_{touch} \cdot 1) \cdot exp(\beta_{sex} \cdot 1) = exp(\beta_0 + \beta_{touch} + \beta_{sex})$$
$$r_{touching\ a\ female} = exp(\beta_0) \cdot exp(\beta_{touch} \cdot 1) \cdot exp(\beta_{sex} \cdot 0) = exp(\beta_0 + \beta_{touch})$$

For example, if $\beta_0 = 0.7$, $\beta_{touch} = 0.1$ and $\beta_{sex} = 1.0$, the neuron is slightly increased during social touch with females and strongly increased during social touch with males:

$$r_{touching\ a\ male} = exp(\beta_0 + \beta_{touch} + \beta_{male}) = exp(0.7 + 0.1 + 1.0) = 6.05Hz$$
$$r_{touching\ a\ female} = exp(\beta_0 + \beta_{touch}) = exp(0.7 + 0.1) = 2.23Hz$$
$$r_{base} = exp(\beta_0) = exp(0.7) = 2.01Hz$$

As another example, if $\beta_0 = 1.3$, $\beta_{touch} = 1.2$ and $\beta_{sex} = -1.2$, the neuron is strongly increased during social touch with females and not modulated at all during social touch with males:

$$r_{touching\ a\ male} = exp(\beta_0 + \beta_{touch} + \beta_{male}) = exp(1.3 + 1.2 - 1.2) = 3.67Hz$$
$$r_{touching\ a\ female} = exp(\beta_0 + \beta_{touch}) = exp(1.3 + 1.2) = 12.18Hz$$
$$r_{base} = exp(\beta_0) = exp(1.3) = 3.67Hz$$

Supplementary Note 2: *Full specification of statistical models plotted in Fig. 4d & Fig. S5b*

Full data and Matlab code required to fit and plot models are provided as supplementary data.

Vibrissa motor cortex - Generalized linear mixed-effects model fit by PL

Model information:

Number of observations	292
Fixed effects coefficients	4
Random effects coefficients	6
Covariance parameters	2
Distribution	Normal
Link	Identity
FitMethod	MPL

Formula:

```
fold_male ~ 1 + own_sex*fold_female + (1 | own_name)
```

Model fit statistics:

AIC	BIC	LogLikelihood	Deviance
398.79	420.85	-193.39	386.79

Fixed effects coefficients (95% CIs):

Name	Estimate	SE	tStat	DF	pValue	Lower	Upper
'(Intercept)'	-0.035103	0.055253	-0.63531	288	0.52573	-0.14385	0.073648
'own_sex_f'	-0.071701	0.095732	-0.74898	288	0.45448	-0.26012	0.11672
**'fold_female'**	**0.37866**	**0.056633**	**6.6862**	**288**	**1.1872e-10**	**0.26719**	**0.49013**
**'own_sex_f:fold_female'**	**0.22373**	**0.085462**	**2.6179**	**288**	**0.0093155**	**0.05552**	**0.39194**

Random effects covariance parameters:
Group: own_name (6 Levels)

Name1	Name2	Type	Estimate
'(Intercept)'	'(Intercept)'	'std'	0.074526

Group: Error

Name	Estimate
'sqrt(Dispersion)'	0.4658

Somatosensory cortex - Generalized linear mixed-effects model fit by PL

Model information:

Number of observations	369
Fixed effects coefficients	4
Random effects coefficients	13
Covariance parameters	2
Distribution	Normal
Link	Identity
FitMethod	MPL

Formula:

```
fold_male ~ 1 + own_sex*fold_female + (1 | own_name)
```

Model fit statistics:

AIC	BIC	LogLikelihood	Deviance
883.47	906.94	-435.74	871.47

Fixed effects coefficients (95% CIs):

Name	Estimate	SE	tStat	DF	pValue	Lower	Upper
'(Intercept)'	0.049988	0.060706	0.82345	365	0.41079	-0.069389	0.16936
'own_sex_f'	-0.0095401	0.083752	-0.11391	365	0.90937	-0.17424	0.15516
**'fold_female'**	**0.65831**	**0.060194**	**10.936**	**365**	**2.8566e-24**	**0.53994**	**0.77668**
**'own_sex_f:fold_female'**	**-0.35135**	**0.097624**	**-3.5991**	**365**	**0.00036346**	**-0.54333**	**-0.15938**

Random effects covariance parameters:
Group: own_name (13 Levels)

Name1	Name2	Type	Estimate
'(Intercept)'	'(Intercept)'	'std'	5.7193e-05

Group: Error

Name	Estimate
'sqrt(Dispersion)'	0.78814

Auditory cortex - Generalized linear mixed-effects model fit by PL

Model information:

Number of observations	236
Fixed effects coefficients	4
Random effects coefficients	9
Covariance parameters	2
Distribution	Normal
Link	Identity
FitMethod	MPL

Formula:
    fold_male ~ 1 + own_sex*fold_female + (1 | own_name)

Model fit statistics:

AIC	BIC	LogLikelihood	Deviance
252.96	273.74	-120.48	240.96

Fixed effects coefficients (95% CIs):

Name	Estimate	SE	tStat	DF	pValue	Lower	Upper
'(Intercept)'	-0.020294	0.053825	-0.37703	232	0.7065	-0.12634	0.085755
'own_sex_f'	-0.013834	0.062491	-0.22137	232	0.825	-0.13696	0.10929
**'fold_female'**	**0.61289**	**0.083716**	**7.321**	**232**	**4.0122e-12**	**0.44795**	**0.77783**
'own_sex_f:fold_female'	-0.17223	0.12944	-1.3306	232	0.18463	-0.42726	0.082797

Random effects covariance parameters:
Group: own_name (9 Levels)

Name1	Name2	Type	Estimate
'(Intercept)'	'(Intercept)'	'std'	4.6881e-06

Group: Error

Name	Estimate
'sqrt(Dispersion)'	0.40316

Cingulate cortex - Generalized linear mixed-effects model fit by PL

Model information:

Number of observations	94
Fixed effects coefficients	4
Random effects coefficients	7
Covariance parameters	2
Distribution	Normal
Link	Identity
FitMethod	MPL

Formula:
    fold_male ~ 1 + own_sex*fold_female + (1 | own_name)

Model fit statistics:

AIC	BIC	LogLikelihood	Deviance
155.45	170.71	-71.725	143.45

Fixed effects coefficients (95% CIs):

Name	Estimate	SE	tStat	DF	pValue	Lower	Upper
**'(Intercept)'**	**-0.14633**	**0.059668**	**-2.4525**	**90**	**0.016116**	**-0.26487**	**-0.027793**
'own_sex_m'	-0.063485	0.16651	-0.38127	90	0.7039	-0.39429	0.26732
'fold_female'	0.011907	0.1134	0.105	90	0.91661	-0.21338	0.23719
'own_sex_m:fold_female'	0.28014	0.31106	0.90057	90	0.37022	-0.33785	0.89812

Random effects covariance parameters:
Group: own_name (7 Levels)

Name1	Name2	Type	Estimate
'(Intercept)'	'(Intercept)'	'std'	3.5072e-05

Group: Error

Name	Estimate
'sqrt(Dispersion)'	0.51897

Prelimbic cortex - Generalized linear mixed-effects model fit by PL

Model information:

Number of observations	136
Fixed effects coefficients	4
Random effects coefficients	5
Covariance parameters	2
Distribution	Normal
Link	Identity
FitMethod	MPL

Formula:

```
fold_male ~ 1 + own_sex*fold_female + (1 | own_name)
```

Model fit statistics:

AIC	BIC	LogLikelihood	Deviance
96.198	113.67	-42.099	84.198

Fixed effects coefficients (95% CIs):

Name	Estimate	SE	tStat	DF	pValue	Lower	Upper
'(Intercept)'	0.020633	0.033138	0.62265	132	0.53459	-0.044916	0.086183
'own_sex_m'	-0.029437	0.064418	-0.45696	132	0.64845	-0.15686	0.097989
'fold_female'	0.15258	0.079862	1.9105	132	0.058233	-0.0053957	0.31055
'own_sex_m:fold_female'	0.35691	0.21527	1.658	132	0.099699	-0.068914	0.78273

Random effects covariance parameters:

Group: own_name (5 Levels)

Name1	Name2	Type	Estimate
'(Intercept)'	'(Intercept)'	'std'	4.4077e-06

Group: Error

Name	Estimate
'sqrt(Dispersion)'	0.32976
