# Supplementary material for: Highly structured, partner-sex- and subject-sex-dependent cortical responses during social facial touch
Source: Nat Commun. 2019 Oct 11;10:4634. doi: 10.1038/s41467-019-12511-z (PMC6789031; doi:10.1038/s41467-019-12511-z)
Supplement: Supplementary file 3 — Reporting Summary [file 41467_2019_12511_MOESM3_ESM.pdf]

nature research

Corresponding author(s): Christian Ebbesen

Last updated by author(s): Aug 30, 2019

Reporting Summary

Nature Research wishes to improve the reproducibility of the work that we publish. This form provides structure for consistency and transparency in reporting. For further information on Nature Research policies, seeAuthors & Referees and theEditorial Policy Checklist .

Statistics

For all statistical analyses, confirm that the following items are present in the figure legend, table legend, main text, or Methods section.

n/a	Confirmed	
☐	☒	The exact sample size (*n*) for each experimental group/condition, given as a discrete number and unit of measurement
☐	☒	A statement on whether measurements were taken from distinct samples or whether the same sample was measured repeatedly
☐	☒	The statistical test(s) used AND whether they are one- or two-sided<br>*Only common tests should be described solely by name; describe more complex techniques in the Methods section.*
☐	☒	A description of all covariates tested
☐	☒	A description of any assumptions or corrections, such as tests of normality and adjustment for multiple comparisons
☐	☒	A full description of the statistical parameters including central tendency (e.g. means) or other basic estimates (e.g. regression coefficient) AND variation (e.g. standard deviation) or associated estimates of uncertainty (e.g. confidence intervals)
☐	☒	For null hypothesis testing, the test statistic (e.g. *F*, *t*, *r*) with confidence intervals, effect sizes, degrees of freedom and *P* value noted<br>*Give P values as exact values whenever suitable.*
☒	☐	For Bayesian analysis, information on the choice of priors and Markov chain Monte Carlo settings
☐	☒	For hierarchical and complex designs, identification of the appropriate level for tests and full reporting of outcomes
☐	☒	Estimates of effect sizes (e.g. Cohen's *d*, Pearson's *r*), indicating how they were calculated

Our web collection on statistics for biologists contains articles on many of the points above.

Software and code

Policy information about availability of computer code

Data collection	Neural data was collected using Neuralynx Cheetah software, video data was collected using StreamPix and custom recording software
Data analysis	Data analysis was done in Matlab

For manuscripts utilizing custom algorithms or software that are central to the research but not yet described in published literature, software must be made available to editors/reviewers. We strongly encourage code deposition in a community repository (e.g. GitHub). See the Nature Research guidelines for submitting code & software for further information.

Data

Policy information about availability of data

All manuscripts must include a data availability statement. This statement should provide the following information, where applicable:

- Accession codes, unique identifiers, or web links for publicly available datasets
- A list of figures that have associated raw data
- A description of any restrictions on data availability

The full raw data from four example neurons (the neurons shown in Figure 3), as well as the Matlab code used to fit and analyze the statistical models and generate plots in Figure 3 is avail-able as supplementary material. Matlab code and a table including the response magnitudes from the fitted 'sex-touch' models, the sex and identity of the subject animal (i.e. all the data needed to reproduce Figure 4 and reproduce the statistical modeling) is available as supplementary materi-al. The full raw dataset is available upon request to the corresponding author.

Field-specific reporting

Please select the one below that is the best fit for your research. If you are not sure, read the appropriate sections before making your selection.

Life sciences study design

All studies must disclose on these points even when the disclosure is negative.

Sample size	Neuons were recorded throughout the cortical column, with a majority of neurons in the deep layers. The laminar distribution was (S1/VMC/PrL/ACC/A1): Layer 6: 32/16/39/31/46, layer 5b: 88/153/70/17/100, layer 5a: 42/37/33/47/63, layer 4: 56/0/0/0/19, layer 2/3: 37/90/0/0/4, layer uncertain: 129/0/0/0/7.
Data exclusions	To avoid biasing the regression fit by the few cells with extremely low firing rates (indicated by circles in Figure S4A), we removed neurons with a more than 32-fold increase/decrease from the fit in Figure 5b.
Replication	No replications.
Randomization	Animals were randomly implanted, we did no pre-selection of animals to assign brain areas.
Blinding	Video data was annotated blind to the neural data.

Reporting for specific materials, systems and methods

We require information from authors about some types of materials, experimental systems and methods used in many studies. Here, indicate whether each material, system or method listed is relevant to your study. If you are not sure if a list item applies to your research, read the appropriate section before selecting a response.

Materials & experimental systems

n/a	Involved in the study
☒	☐ Antibodies
☒	☐ Eukaryotic cell lines
☒	☐ Palaeontology
☐	☒ Animals and other organisms
☒	☐ Human research participants
☒	☐ Clinical data

Methods

n/a	Involved in the study
☒	☐ ChIP-seq
☒	☐ Flow cytometry
☒	☐ MRI-based neuroimaging

Animals and other organisms

Policy information about studies involving animals; ARRIVE guidelines recommended for reporting animal research

Laboratory animals	Rats (Wistar) were purchased from Janvier Labs (Le Genest-Saint-Isle, France). Rats presented as partner animals were housed socially in same-sex cages, and post-surgery implanted ani-mals were housed in single animal cages. All animals were kept on a 12h:12h reversed light/dark cycle and all experiments were performed in the animals' dark phase. Rats had ad libitum access to food and water.
Wild animals	No wild animals.
Field-collected samples	No field samples.
Ethics oversight	All experimental procedures were performed according to German animal welfare law under the supervision of local ethics committees.

Note that full information on the approval of the study protocol must also be provided in the manuscript.
