# Supplementary material for: Highly structured, partner-sex- and subject-sex-dependent cortical responses during social facial touch
Source: Nat Commun. 2019 Oct 11;10:4634. doi: 10.1038/s41467-019-12511-z (PMC6789031; doi:10.1038/s41467-019-12511-z)
Supplement: Supplementary file 4 — Description of Additional Supplementary Files [file 41467_2019_12511_MOESM4_ESM.pdf]

Description of Additional Supplementary Files

File Name: Supplementary Software 1
Description: The subfolder 'Figure_1_and_3' contains the full raw data from four example neurons (the neurons shown in Figure 3), as well as the Matlab code used to fit and analyze the statistical models and generate plots, such as shown in Figure 1 and 3. The subfolder 'Figure 4_and_5' contains Matlab code and a table including the response magnitudes from the fitted 'sextouch' models, the sex and identity of the subject animal (i.e. all the data needed to reproduce Figure 4-5 and reproduce the statistical modeling). The subfolder 'Figure_6' contains all python code required to run the simulations shown in Figure 6, and the python code to analyze the simulated data and generate the figure panels.
