# Supplementary figures and images for: Highly structured, partner-sex- and subject-sex-dependent cortical responses during social facial touch
Source: Nat Commun. 2019 Oct 11;10:4634. doi: 10.1038/s41467-019-12511-z (PMC6789031; doi:10.1038/s41467-019-12511-z)

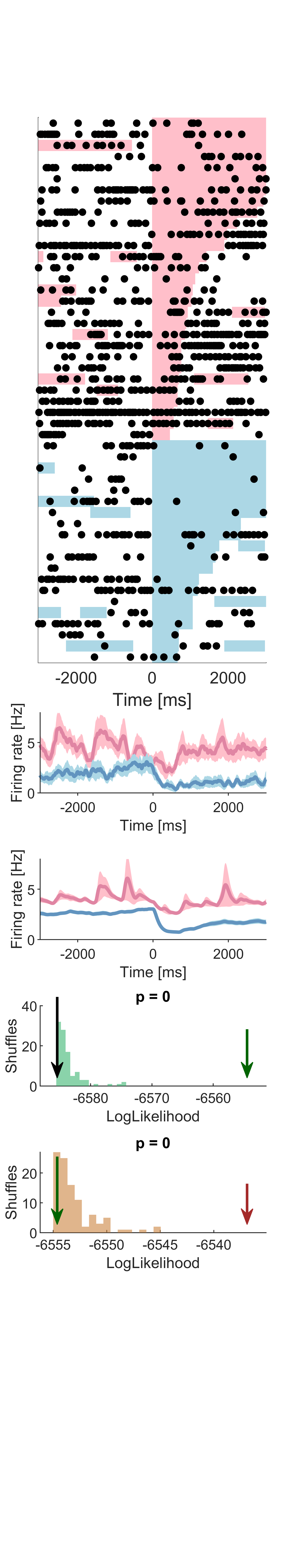

Supplement: Supplementary file 5 — Supplementary Software 1 [file 41467_2019_12511_MOESM5_ESM.zip › Ebbesen2019_code_and_data/Figure_1_and_3/example_plot_cebbesen_462.png]

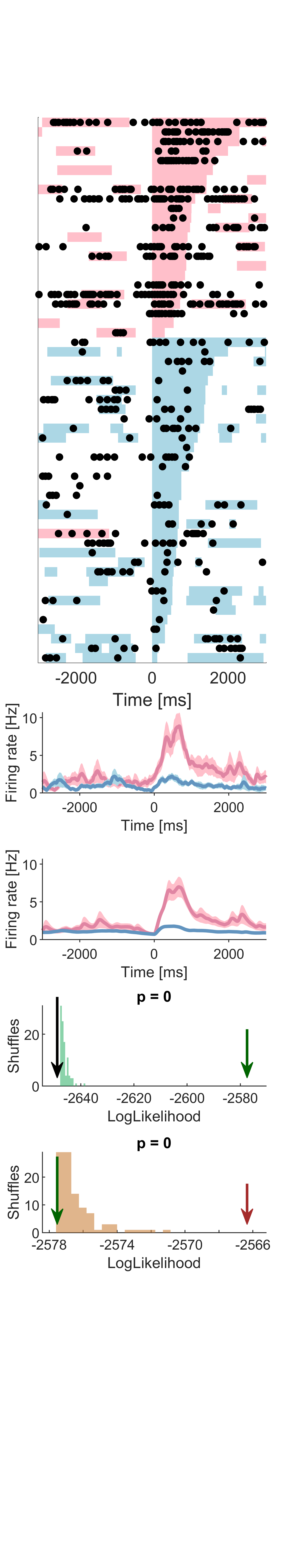

Supplement: Supplementary file 5 — Supplementary Software 1 [file 41467_2019_12511_MOESM5_ESM.zip › Ebbesen2019_code_and_data/Figure_1_and_3/example_plot_cebbesen_687.png]

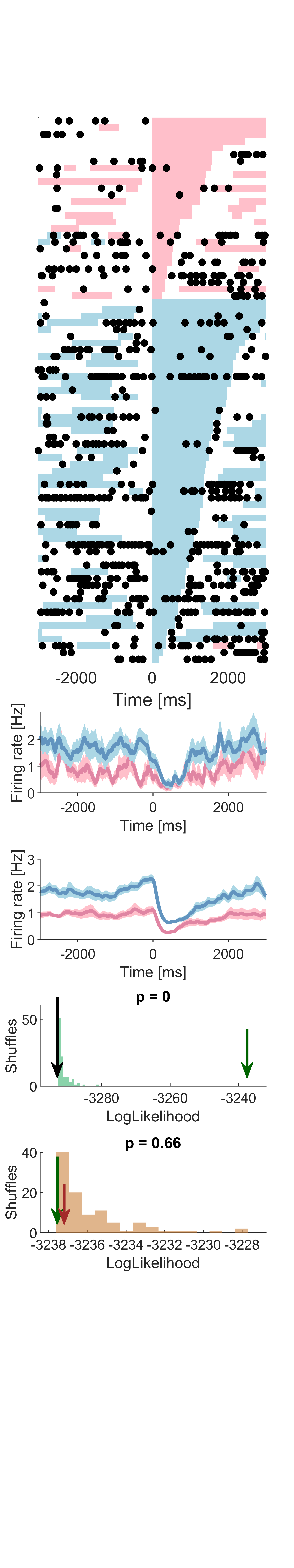

Supplement: Supplementary file 5 — Supplementary Software 1 [file 41467_2019_12511_MOESM5_ESM.zip › Ebbesen2019_code_and_data/Figure_1_and_3/example_plot_cebbesen_759.png]

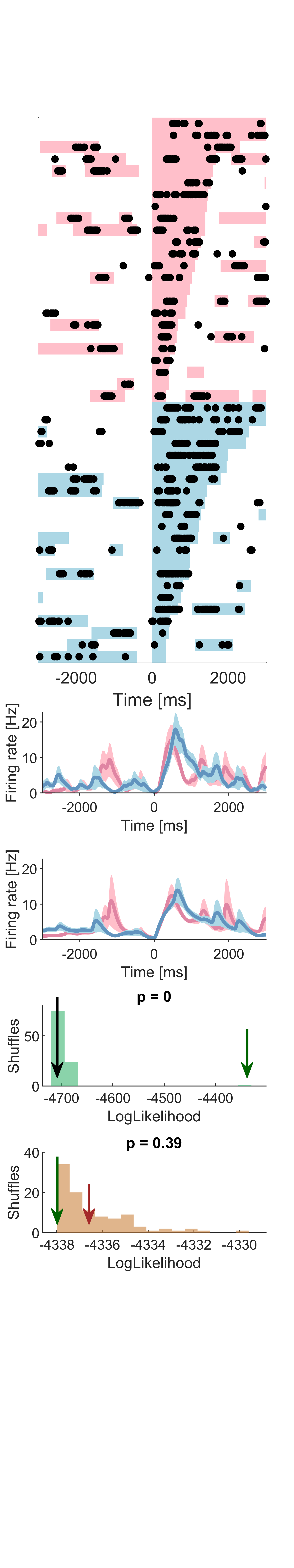

Supplement: Supplementary file 5 — Supplementary Software 1 [file 41467_2019_12511_MOESM5_ESM.zip › Ebbesen2019_code_and_data/Figure_1_and_3/example_plot_evgeny_81.png]

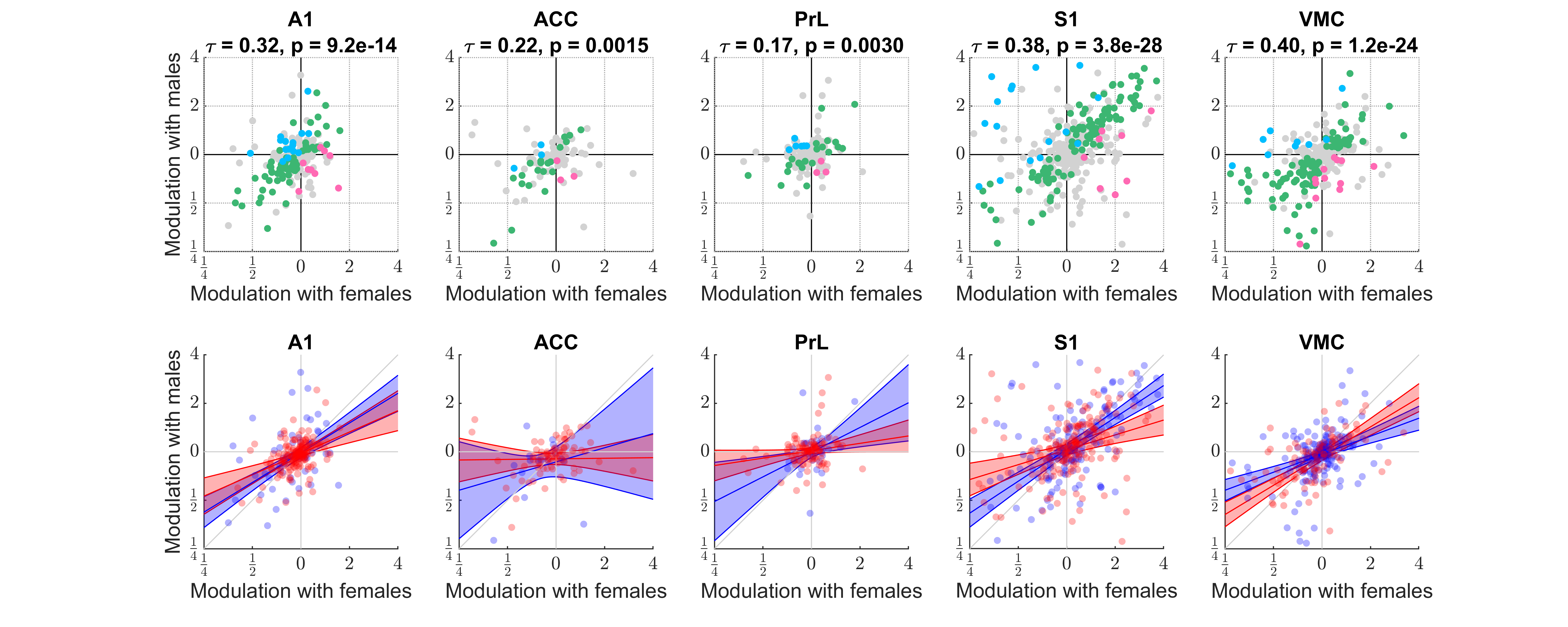

Supplement: Supplementary file 5 — Supplementary Software 1 [file 41467_2019_12511_MOESM5_ESM.zip › Ebbesen2019_code_and_data/Figure_4_and_5/example_clouds.png]

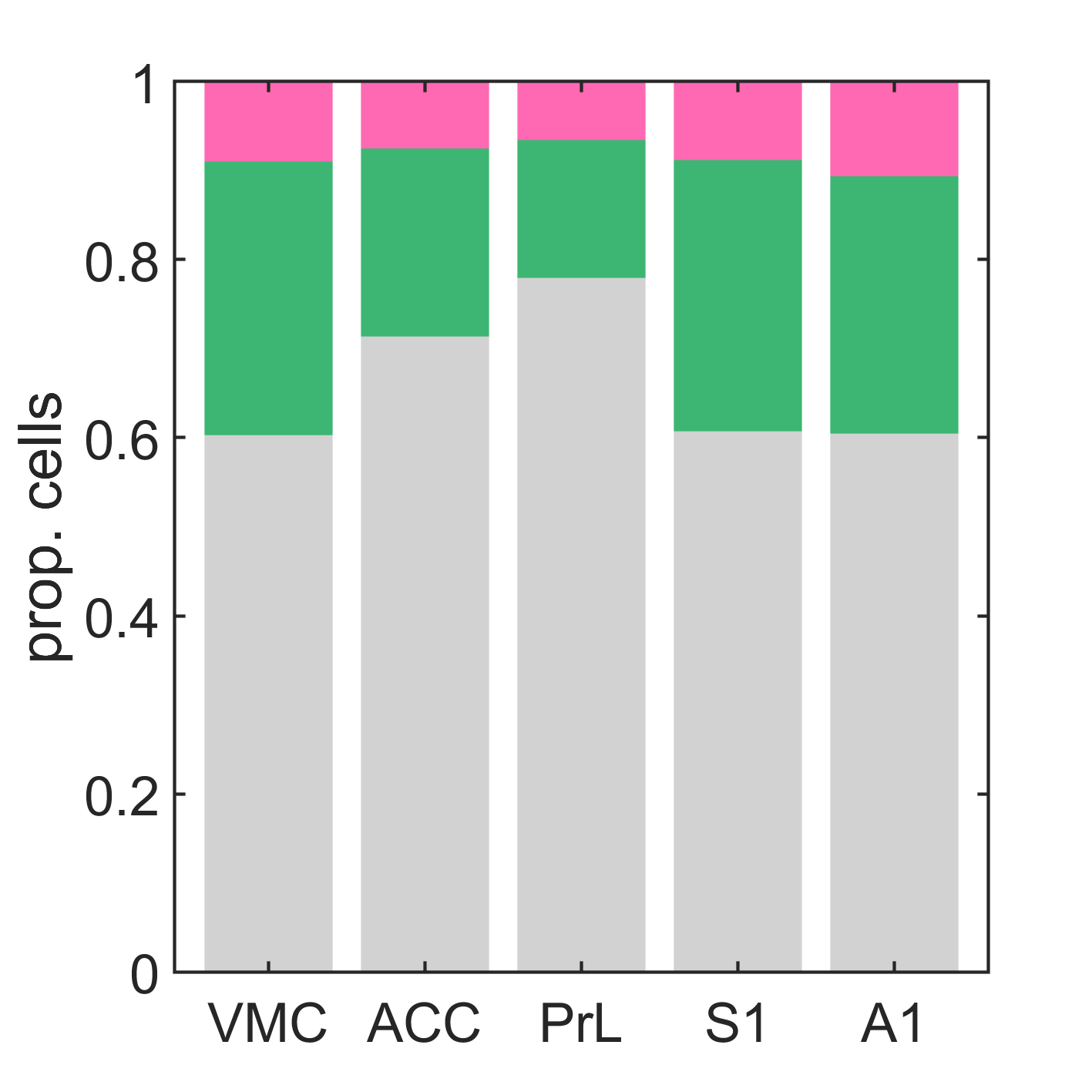

Supplement: Supplementary file 5 — Supplementary Software 1 [file 41467_2019_12511_MOESM5_ESM.zip › Ebbesen2019_code_and_data/Figure_4_and_5/example_counts.png]
